# Addressing inequities in maternal health among women living in communities of social disadvantage and ethnic diversity

**DOI:** 10.1186/s12889-021-10182-4

**Published:** 2021-01-21

**Authors:** Cristina Fernandez Turienzo, Mary Newburn, Agnes Agyepong, Rachael Buabeng, Amy Dignam, Clotilde Abe, Leah Bedward, Hannah Rayment-Jones, Sergio A. Silverio, Abigail Easter, Lauren E. Carson, Louise M. Howard, Jane Sandall, On behalf of the NIHR ARC South London Maternity and Perinatal Mental Health Research and Advisory Teams, Anna Horn

**Affiliations:** 1 Department of Women and Children’s Health, Faculty of Life Science and Medicine, King’s College London, London, UK; 2 Maternity Voices Partnership, Guys and St Thomas’ NHS Foundation Trust, London, UK; 3 Mummy’s Day Out, London, UK; 4 Maternity Voices Partnership, Chelsea and Westminster Hospital NHS Foundation Trust, London, UK; 5 Prosperitys Trust and Five X More, London, UK; 6 Recent service user, London, UK; 7 Department of Health Service and Population Research, Institute of Psychiatry, Psychology & Neuroscience, King’s College London, London, UK; 8 Department of Psychological Medicine, Institute of Psychiatry, Psychology & Neuroscience, King’s College London, London, UK

**Keywords:** Public health, Maternity research, Community involvement, Patient and public involvement and engagement

## Abstract

The response to the coronavirus outbreak and how the disease and its societal consequences pose risks to already vulnerable groups such those who are socioeconomically disadvantaged and ethnic minority groups. Researchers and community groups analysed how the COVID-19 crisis has exacerbated persisting vulnerabilities, socio-economic and structural disadvantage and discrimination faced by many communities of social disadvantage and ethnic diversity, and discussed future strategies on how best to engage and involve local groups in research to improve outcomes for childbearing women experiencing mental illness and those living in areas of social disadvantage and ethnic diversity. Discussions centred around: access, engagement and quality of care; racism, discrimination and trust; the need for engagement with community stakeholders; and the impact of wider social and economic inequalities. Addressing biomedical factors alone is not sufficient, and integrative and holistic long-term public health strategies that address societal and structural racism and overall disadvantage in society are urgently needed to improve health disparities and can only be implemented in partnership with local communities.

## Background

The COVID-19 pandemic, and the wider political and social response have brought health and social inequalities into sharp focus in the UK (and elsewhere) [1]. Black, Asian and minority ethnic groups and most deprived populations are at increased risk of virus exposure and pre-existing health conditions put them at increased morbidity and mortality if they contract the virus [2]. In pregnant women, over half of those recently admitted to hospital with the infection were from Black, Asian and minority ethnic groups [3]. Pre-pandemic enquiries of all maternal and perinatal deaths found these women and/or those living in deprived areas were more likely to experience mental illness, to lose their babies or die during or after their pregnancy [4, 5]. The COVID-19 crisis has exacerbated persisting vulnerabilities, socio-economic and structural disadvantage and discrimination faced by many communities of social disadvantage and ethnic diversity [6].

Measures introduced to control spread of the virus (e.g. lockdown, social distancing, suspension of non-urgent NHS services) have added social and economic burdens disproportionately on those already experiencing inequality, and the ensuing economic recession will worsen inequalities in maternal, child and family health for years to come [1, 7, 8]. For example, pregnant women from some Black, Asian and minority ethnic groups, and the most socially disadvantaged backgrounds are more likely to suffer severe maternal morbidities and experience poor infant outcomes such as stillbirth and preterm birth, which can led to significant problems and disabilities in the future [9, 10]. This can have major implications for the child, the mother and the family’ health and wellbeing, whose levels of vulnerability are likely to be exacerbated during the recession, affecting experiences and already limited access to specialist care, education and family building and support networks [11, 12]. Therefore, the time for action is now. Supporting community participatory research, in which community stakeholders’ partner with researchers to understand the interplay of complex and multiple factors (physical, mental and social, as well as cultural, structural, economic, religious, commercial), is crucial to inform public health strategies designed to reduce inequities among Black, Asian and minority ethnic and socially disadvantaged groups [6].

The National Institute for Health Research (NIHR) has provided £135 million for 5-years funding to 15 Applied Research Collaborations (ARCs) across the UK, establishing partnerships between NHS providers, universities, charities, local authorities and other organisations. The ARCs aim to support research that improves outcomes, quality, delivery, and efficiency of health services for patients and the public by directly responding to, and meeting, the needs of local populations and local health and care systems [13]. Our maternity and perinatal mental health research theme, part of ARC South London, plans to address poorer outcomes for childbearing women experiencing mental illness and those living in areas of social disadvantage and ethnic diversity, to reduce health inequities and have a positive impact on health and wellbeing of women and families in the local community. There is a focus on people-centre and place-based models of maternity care that integrate health and care by shifting the way services are funded, managed, and delivered “from health systems designed around diseases and health institutions towards health systems designed for people and communities” [14, 15].

## Community engagement

We consider Patient and Public Involvement and Engagement (PPIE) central to ARC maternal health research [16] and we have begun our programme of work by engaging women involved in local communities in South London. Their experiences and insights into maternal and perinatal mental health can help to make our research more relevant to the needs and priorities of the local population. Since people living in the most deprived areas and those from Black, Asian and minority ethnic groups are least likely to be involved in research [17], it is particularly important to engage and involve community leaders, social entrepreneurs, local maternity voices partnerships and charities with established connections.

As a starting point for involvement in research studies and in co-design of a PPIE strategy, we held two online engagement events in June 2020 to discuss current issues on maternal health in South London and start building collaborations with local communities [18]. COVID-19 restrictions meant that face-to-face meetings were not possible, so we held the events online using video conferencing. A total of 13 London-based service user representatives, seven researchers, two PPIE leads, and three other contributors attended. The service users and contributors identified themselves as Black, Asian, White and mixed ethnicity women who, after introductions, talked freely about their personal and community knowledge and insights. The 60–90 min events, recorded with the permission of participants, were friendly and informal; and discussion was prompted occasionally by open questions. The main focus of this commentary paper is to present a summary of the issues raised from both engagement events to raise awareness of what is important for Black, Asian and minority ethnic communities and those who experience social disadvantage at this point in time, so as to address inequalities in maternity research and subsequently better inform policy makers.

## Key themes

Four themes were identified: access, engagement and quality of care; racism, discrimination and trust; community stakeholders; and wider social and economic inequalities.

### Access, engagement and quality of care

- The concept of a postcode lottery was raised by attendees who have lived in both deprived and wealthier areas, acknowledging that equitable quality maternity care is not a current reality, though this may not be recognised by women if they have not experienced better quality care somewhere else.
- Perceptions of little or no representation of Black, Asian and minority ethnic and disadvantaged groups in maternity and childbirth networks and safe spaces reduced engagement in services, education and social support.
- The perinatal period was considered a missed opportunity for connecting women with services; more responsive, user-focused, services are still needed.
- The group valued continuity of care when it happened, but they felt most women received fragmented care (particularly in childbirth), often having to repeat their stories to different care providers which was challenging particularly for disadvantaged women and those with experience of abuse or trauma.
- Maternal mental health problems are more common among Black, Asian and minority ethnic and disadvantaged groups, yet the group felt they were often forgotten during pregnancy (particularly those of mild-moderate severity).
- It was noted that different communities understand and talk about mental health in different ways, and in certain communities mental illness is rarely spoken about because of social stigma or mistaken beliefs that it cannot be treated. This can discourage people from talking about it, seeking help or engaging with services.
- The effects of COVID-19 on access to maternity services and quality of care were also raised, including reduced visits and face to face contact, lack of choices, lack of personalised care and continuity, delay in emergency procedures, closure of community centres, effect of self-isolation on mental health and impact of domestic violence under lockdown.

### Racism, discrimination and trust

- Attendees pointed to racism and discrimination experienced, particularly by Black, Asian and minority ethnic and socially disadvantaged groups, as a root cause of poorer and less respectful treatment. It was felt that systemic racism and discrimination could be seen in attitudes and behaviours in healthcare institutions.
- Some Black members of the group raised as a suggestion for further research the potential value of exploring the beliefs, experiences and expectations of Black care providers and family members and how these might influence health inequalities in access to services and interactions between maternity staff and service users.
- It was also noted that fear or lack of trust in services could result in reluctance to seek prompt care, and more subtlety, a sense that services were ‘other’ and culturally insensitive. This reduced motivation to attend, especially in the face of everyday demands and conflicting pressures. Strategies to develop cultural awareness and build respect among health care providers and systems are needed.
- The importance of being listened to, respected, and treated as a unique individual was emphasised in multiple occasions.

### Community stakeholders

- A lot was said about the importance of advocacy and community groups for women’s and communities’ education and empowerment, as well as the power of social media as a campaign and communication tool.
- The service users highlighted the importance of involving community groups in selecting relevant research questions. Also, the use of participatory methods was recommended as a way to engage with disadvantaged communities and enable them to have a voice. This can involve training women who are socially disadvantaged or Black, Asian and minority ethnic as peer researchers and paying them to co-facilitate events to gather qualitative data [19].
- Carrying out engagement activities, workshops, focus groups and interviews in neutral locations (non-institutional settings such as universities and hospitals) was advised.
- The value of establishing relationships with local community leaders and networks to enhance the depth of reach into Black, Asian and minority ethnic communities was emphasised repeatedly.

### Wider social and economic inequalities

- The necessity to consider wider factors in addition to ethnicity was also raised. As many Black, Asian and minority ethnic and disadvantaged women face multiple social complexities, it is important to understand how they can feel safe, what are their circles of support (and what happens when those are not there), and how they experience health problems and services provision.
- The term “weathering” was introduced by one of the attendees, originally proposed to understand accelerated health deterioration observed among African American women, as a result of cumulative exposure to adversity and socio-economic disadvantage (e.g., more poverty, lower education, higher unemployment).
- The group emphasised that addressing biomedical factors alone is not sufficient. They wanted integrated and holistic long-term public health strategies that address societal and structural racism and overall disadvantage in society which, they said, can only be implemented in partnership with local communities.

## Summary and conclusion

The views and observations of service users and the research literature suggest that policymakers, the NHS, and local government need to take a community place-based approach to reducing maternal health inequalities, and to work in partnership with community leaders. Charities and online networks can act as a source of information and introduction to many Black, Asian and minority ethnic and socially disadvantaged groups. We aim to work together throughout the ARC programme, agreeing a PPIE strategy and meeting regularly. We will focus on opportunities to co-produce maternity research, and will seek additional funding and opportunities for co-production training, including participatory appraisal research.

The poorer health outcomes for some Black, Asian and minority ethnic pregnant women have been acknowledged by the government and Royal Colleges [20, 21]. The NHS Long Term Plan additional funding will be available to support the provision of continuity of care with accelerated implementation for these women and those from the most deprived neighbourhoods [22]. We believe, this may make a difference, but will not be sufficient without broader measured to address inequalities and community engagement. The perceptions and insights of local communities are key to researching lived experiences and implementing measures to address inequalities and overall health improvement.

## Data Availability

All data analysed are included within this published article.
